# The Relationship Between Clinical Imaging and Neurobehavioral Assessment in Posthemorrhagic Ventricular Dilation of Prematurity

**DOI:** 10.3389/fphys.2019.00064

**Published:** 2019-02-11

**Authors:** Rebecca A. Dorner, Bruno P. Soares, Shenandoah Robinson, Marilee C. Allen, Jamie Perin, Vera Joanna Burton

**Affiliations:** ^1^Neonatology, Johns Hopkins Hospital, Baltimore, MD, United States; ^2^Neurosciences Intensive Care Nursery, Johns Hopkins Hospital, Baltimore, MD, United States; ^3^Pediatric Radiology and Pediatric Neuroradiology, Johns Hopkins Hospital, Baltimore, MD, United States; ^4^Pediatric Neurosurgery, Johns Hopkins Hospital, Baltimore, MD, United States; ^5^Biostatistics, Epidemiology, and Data Management Core, Johns Hopkins Hospital, Baltimore, MD, United States; ^6^Neurology and Developmental Medicine, Kennedy Krieger Institute, Baltimore, MD, United States; ^7^Department of Neurology, The Johns Hopkins School of Medicine, Baltimore, MD, United States

**Keywords:** intraventricular hemorrhage, prematurity, ventriculomegaly, hydrocephalus, development, neuroimaging

## Abstract

**Introduction:** Neonatal intraventricular hemorrhage (IVH) and subsequent posthemorrhagic ventricular dilation and hydrocephalus of prematurity are associated with brain injury and neurodevelopmental impairment in the preterm population. Neuroimaging assesses cerebral injury and guides neurosurgical intervention; however, the relationship of head ultrasound (HUS) and magnetic resonance imaging (MRI) parameters to neonatal exams in this group has not been well described. The NICU Network Neurobehavioral Scale (NNNS) is a reproducible, highly reliable battery with motor and cognitive domain scores.

**Objective:** To evaluate the relationship between neonatal neurobehavioral findings on the NNNS and measures of ventricular dilation and associated brain injury on HUS and MRI.

**Materials and Methods:** Neonates with IVH and ventricular dilatation with and without posthemorrhagic hydrocephalus were enrolled. NNNS exams were performed at approximately term age equivalent. HUS indices were measured on the last HUS before initial neurosurgical procedure or that with worst ventriculomegaly if no intervention. The posterior fossa was assessed with MRI at term. Descriptive statistics including medians, interquartile ranges, means, and percentages were performed. Correlations were estimated using Pearson's method.

**Results:** 28 patients had NNNS and HUS, and 18 patients also had an MRI. Ventricle size measures for the cohort were significantly above normal. Motor and cognitive subscores on the NNNS exam varied from established baseline scores for postmenstrual age. Children who required neurosurgical intervention had higher ventricle/brain ratios and worse NNNS habituation scores. Ventricle sizes were modestly correlated with motor abnormalities (0.24–0.59); larger anterior horn width correlated with nonoptimal reflexes, hypertonicity and hypotonicity. Ventricle sizes were modestly correlated with cognitive scores (−0.44 to 0.27); larger ventricular index correlated with worse attention. Periventricular hemorrhagic infarction correlated with worse habituation.

**Conclusion:** For this cohort of preterm infants with IVH, surgical intervention for posthemorrhagic hydrocephalus correlated with both larger degrees of ventriculomegaly and worse NNNS exams. Findings on both HUS and MRI correlated with motor and cognitive abnormalities on neonatal neurobehavioral exam, suggesting that larger neonatal ventricle sizes and white matter injury have detectable correlates on exam. The NNNS exam provides important additional information when assessing posthemorrhagic ventricular dilation and hydrocephalus of prematurity.

## Background

Despite advances in neonatal care, intraventricular hemorrhage (IVH) remains a serious complication of prematurity occurring in up to 30% of premature infants. It is estimated that one-third to half of infants with severe IVH (grades 3 or 4) develop posthemorrhagic ventricular dilatation (Alan et al., 2012; Robinson, 2012). Approximately 10% of all neonates with IVH and 20% of infants with severe IVH will need surgical intervention due to posthemorrhagic hydrocephalus (Alan et al., 2012; Robinson, 2012). Preterm children with posthemorrhagic ventricular dilatation, and especially posthemorrhagic hydrocephalus requiring surgical intervention, are at high risk for future neurodevelopmental challenges. Abnormal motor outcomes include a spectrum from cerebral palsy to minor neuromotor dysfunction/developmental coordination disorder (Spittle and Orton, 2014). Other areas of neurodevelopment impacted include: intellectual disability, fine motor coordination problems, memory and executive function deficits, chronic pain, behavior problems, depression and anxiety, attention deficit/hyperactivity disorder and cortical visual impairment (Ment et al., 1999; Brouwer et al., 2008; Roze et al., 2009; Goldstein et al., 2013; Guzzetta et al., 2013; Tsai et al., 2014; Holwerda et al., 2016). Despite these risks, limited data is available regarding observable neurologic abnormalities in the neonatal period in children with ventriculomegaly and hydrocephalus.

The NNNS, or NICU Network Neurobehavioral Scale, is a standardized assessment tool used to measure neurologic integrity and organization (i.e., active and passive tone, reflexes), behavioral and state regulation, and stress/abstinence (Lester et al., 2004). It has strong psychometric qualities and good validity in the assessment of motor and cognitive deficits in the newborn period (Noble and Boyd, 2012) and normative values are available (Tronick et al., 2004). Administration of the exam requires certification after formal instruction and reliability testing. The examination consists of 45 administration and 70 observation items. Summary scores are created for 13 neurobehavioral domains including: habituation, attention, handling, quality of movement, regulation, nonoptimal reflexes, asymmetrical reflexes, stress/abstinence, arousal, hypertonicity, hypotonicity, excitability, and lethargy (Tronick et al., 2004). See Table 1 for descriptions of each summary score (Tronick et al., 2004). NNNS performance at various time points including birth, term age equivalent, and time of neonatal admission discharge, have been independently correlated with later developmental outcomes in children with a variety of high-risk conditions, including neonatal abstinence syndrome(for which it was originally developed) (Lester et al., 2004), premature and low birthweight babies (Lester et al., 2011; El-Dib et al., 2012), term babies with fetal risk factors (Appleton et al., 2016) and more recently children with hypoxic ischemic encephalopathy (Massaro et al., 2015). Importantly, the NNNS is especially equipped to assess early cognitive function and state regulation. Regulation and adaptation to negative stimuli in the NNNS can begin to predict behavioral regulation, “a higher function that would be missed by other routine neurological examinations” (Lester et al., 2011).

**Table 1:** NNNS summary score descriptions (Tronick et al., 2004).

**MOTOR SUBSCORES**	
Excitability	Measure of high levels of motor, state and physiologic reactivity; sum of items for excitable behaviors
Lethargy	Measure of low levels of motor, state and physiologic reactivity; sum of items for lethargic behaviors
Nonoptimal reflexes	Any nonoptimal response to reflex excitation; sum of items for nonoptimal reflexes
Asymmetric reflexes	Any asymmetric response to reflex excitation; sum of items for asymmetric reflexes
Hypertonicity	Hypertonic response in arms, legs, trunk, or in general tone, sum of items for hypertonic indicators
Hypotonicity	Hypotonic response in arms, legs, trunk, or in general tone, sum of items for hypotonic indicators
Quality of movement	Measurement of motor control including smoothness, maturity, lack of startles and tremors; mean of items recoded for good motor control
**COGNITIVE SUBSCORES**	
Habituation	Response decrement to repeated auditory and visual stimuli; mean of items
Attention	Response to animate and inanimate auditory and visual stimuli; mean of items
Handling	Handling strategies used during orientation to maintain alert state; mean number of strategies needed
Regulation	Capacity to organize motor activity, physiology, and state during the examination and to respond to cuddling, consoling, and negative stimuli; mean of items recoded for good regulation
Arousal	Level of arousal including state and motor activity during the examination; mean of items for high arousal
Stress/abstinence	Mean amount of observed stress signs

There are no studies in the literature on NNNS exams in children with posthemorrhagic hydrocephalus of prematurity. The NNNS may help caregivers better understand the infant by providing information about early cognitive and motor function. Additionally, exams can be used serially to recognize subtle changes indicative of worsening neurologic status to guide neuroprotective and rehabilitative interventions.

The aim of our study was to evaluate the association between quantitative measures of neurobehavioral performance in the neonatal period with current gold standard measures of cerebral injury, head ultrasound imaging (HUS) and adjunctive magnetic resonance imaging (MRI). We hypothesized that children with more ventricular dilation, white matter injury, and those requiring intervention for posthemorrhagic hydrocephalus would have worse neonatal neurobehavioral assessments in cognitive and motor domains. Neonatal neurobehavioral exams, if associated with both imaging and later developmental milestones, could function as a bridge between commonly-performed imaging and early milestone attainment.

## Materials and Methods

**Figure 1:**
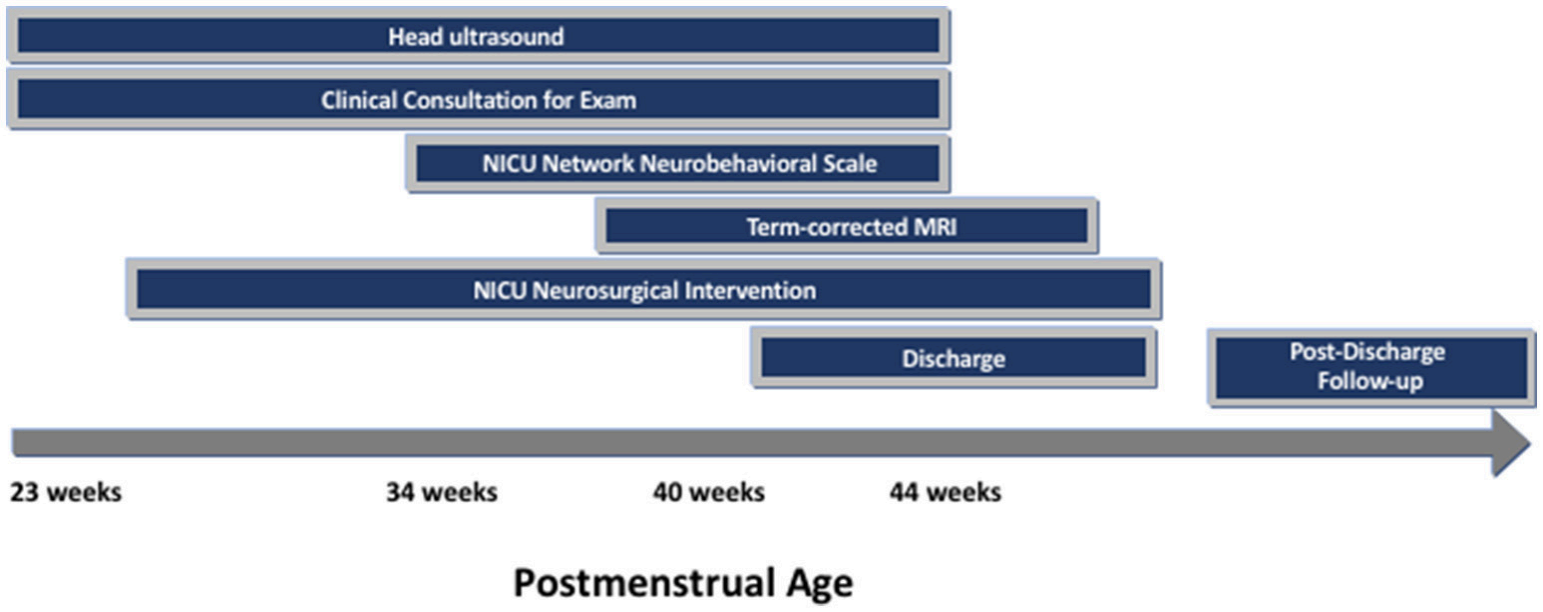
Cohort Timeline of Clinical Milestones, NNNS exam, and Neuroimaging.

Recruitment for this prospective cohort study was conducted from July 1, 2016–July 31, 2018. Eligible patients were preterm infants born in this time frame with IVH and ventricular dilatation, with or without posthemorrhagic hydrocephalus. Babies were either in-born or referred to the Johns Hopkins Hospital Level IIIB and IIIC neonatal intensive care units. Infants were excluded if they had suspected or confirmed genetic anomalies. Infants were identified via neurodevelopmental consultation from the Neurosciences Intensive Care Nursery or by request of the clinical team as part of our routine practice for infants with IVH and ventricular dilatation. After NNNS examination was performed for clinical purposes, parents were approached to consent to join the research cohort. Clinically obtained standard-of-care neuroimaging were recorded in addition to NNNS exam results (see Figure 1). Twenty-Eight eligible infants were enrolled in this time frame, including 88% (16/18) of infants admitted with posthemorrhagic hydrocephalus.

Baseline demographics and comorbidities, including IVH grade, sepsis, necrotizing enterocolitis (NEC), bronchopulmonary dysplasia (BPD) severity (Jobe and Bancalari, 2001) and retinopathy of prematurity (ROP), were recorded (Table 2). Covariates included postmenstrual age at time of NNNS, HUS, and MRI.

**Table 2:** Characteristics of 28 Preterm Neonates with IVH with and without Hydrocephalus (*n* = 28).

Mean gestational age at birth	27.2 weeks (range: 23.4–34.2 weeks)
Mean birthweight	1,130 g (500–3,816 g)
Mean PMA at NNNS Number of exams performed in PMA category	37.2 weeks (32.4–45.1 weeks) < 34 weeks: 3 34.0−34.6 weeks: 5 35.0−35.6 weeks: 3 36.0−36.6 weeks: 4 37+ weeks: 13
Mean PMA at HUS	37.2 weeks (25.6–41.3 weeks)
Mean PMA at MRI	39.5 weeks (33.5–49.2 weeks)
Sex	16 (57%) male, 12 (43%) female
Ethnicity	9 (32%) African-American, 16(57%) Caucasian, 1(4%) Asian, 2 (7%) Hispanic
Adequate prenatal betamethasone^*^	18 (64%)
Grade of Intraventricular hemorrhage	Grade 1: 4 (14%) Grade 2: 3 (11%) Grade 3: 11 (39%) Grade 4 (PVHI): 10 (36%)
Moderate/severe BPD^+^	29 (68%)
Severe ROP (Stage 3 or surgical intervention/biologic medication)	7(25%)
Necrotizing enterocolitis (NEC)^∧^	2 patients Stage 3B, 1 patient Stage 3B after spontaneous intestinal perforation, 1 patient Stage 1B
Treated for culture positive or negative sepsis	20 (71%)
5-min Apgar < 7	11(39%)

*PMA, Postmenstrual Age*.

* *Completed 24 h in-utero after second dose*.

+ *Per NICHD criteria (Jobe and Bancalari, 2001); moderate BPD as O_2_ for ≥28 days plus treatment with < 30% FiO_2_ at 36 weeks' PMA and severe BPD as O_2_ for ≥ 28 days plus ≥ 30% FiO_2_ and/or positive pressure at 36 weeks' PMA*.

∧ *Modified bell staging criteria for NEC (Lee and Polin, 2003)*.

NNNS exams were performed at the closest time point to term age equivalent, ideally no sooner than 34 weeks or less than 10 days from any surgical procedure. Although NNNS exams can be performed in infants from 30 to 48 weeks, we wanted to compare our infant exams to well-established norms at term age equivalent. We chose no sooner than 10 days after a procedure, neurosurgical or otherwise, as we found that with any less time infants were excessively irritable. Per exam criteria, NNNS scores were only performed in medically stable infants with the agreement of the treating medical team. NNNS summary scores were grouped into motor and cognitive categories. Motor subscores include hypertonicity, hypotonicity, excitability, lethargy, nonoptimal reflexes, asymmetrical reflexes, and quality of movement. Cognitive subscores include habituation, attention, handling, regulation, stress/abstinence, and arousal.

The last HUS prior to surgical intervention was selected for measurements as it typically represents the most severe ventricular dilation. If not requiring surgical intervention, the HUS with worst ventriculomegaly was selected. A pediatric neuroradiologist (B.P.S) blinded to clinical course measured the following HUS indices of ventricular size: left and right ventricular index (VI), anterior horn width (AHW), thalamooccipital distance (TOD), and ventricle/brain (V/B) ratio. The left (L) is recorded for each sided measurement for brevity as there was no significant difference amongst right- and left- sided values. Doppler resistive indices (RI) in the anterior cerebral artery, both with and without gentle manual pressure on the transducer, as well as presence of periventricular hemorrhagic infarction (PVHI) and cystic changes were evaluated. The posterior fossa was assessed with MRI; the degree of fourth ventricle dilatation was qualitatively scored (0- none; 1-mild, fourth ventricle compresses vermis only; 2-moderate, compresses dorsal brainstem; 3-severe, compresses ventral brainstem against clivus; 4- massive, fourth ventricle extends superiorly to supratentorial compartment), and the anterior-posterior diameter of the cerebellum from the fastigial point to the posterior vermis (pyramid) was measured. Due to the anatomical distortion of brain parenchyma in the majority of the patients, we were not able to complete typical preterm MRI scoring systems such as the Kidokoro system (Kidokoro et al., 2013). For example, due to the severity of fourth ventricle dilation, cerebellar height and transcerebellar diameter were unable to be completed for the majority of patients. Additionally, HUS measurements of ventricular size agreed with MRI measures, and the sample size of MRIs was more limited than HUS. For this reason, the majority of our statistics analyze HUS measures, with the exception of the fourth ventricle size and anterior-posterior diameter of the cerebellum by MRI. Descriptive statistics including medians, interquartile ranges (IQRs), means, and percentages were used to summarize the cohort. Correlations were estimated using Pearson's method. All analyses were conducted in R version 3.5.0 (R Core Development Team, 2018).

## Results

In total, 28 patients were consented and had the NNNS and HUS performed. Of these 28, 18 patients had the NNNS, HUS and MRI performed. Average gestational age at birth was 27.1(range 23.4–34.2) weeks and average birthweight 1,115 (range 500–3816) g (Table 2). Mean postmenstrual age (PMA) at the time of the HUS was 32.2 weeks, NNNS exam was 37.2 weeks, and MRI was 39.5 weeks (Table 2). Ventricle size measures for the cohort were significantly above normal (Table 3). Motor and cognitive subscores on the NNNS exam (Table 4) varied from established baseline scores for term-corrected age (Lester and Tronick, 2004; Lester et al., 2011; Appleton et al., 2016). The only significant difference between infants with and without MRI was that babies with MRIs were more likely to have a 5 min APGAR score less than 7 (59 vs. 10%, *p* = 0.018).

**Table 3:** Head ultrasound and magnetic resonance imaging parameters.

**Measure**	**Number patients**	**Cohort median (IQR)**	**Normal (Sondhi et al., 2008; Brouwer et al., 2010, 2012; Maunu et al., 2011; Graca et al., 2013; Kidokoro et al., 2013)**
Ventricular index (mm)	28	21.00 (10.75–24.25)	10–13
Anterior horn width (mm)	28	19.00 (8.50–24.25)	Less than 3
Thalamooccipital distance (mm)	28	40.00 (23.75–45.25)	5–25
Ventricle/brain ratio	28	0.52 (0.32–0.60)	Less than 0.35
Resistive Index (with pressure)	28	0.87 (0.78–0.93)	0.5–0.8
Resistive Index (without pressure)	28	0.81 (0.75–0.89)	0.5–0.8
AP diameter cerebellum (mm)	18	14.00 (10.25–17.25)	22–24
4th ventricle dilatation (qualitative score)^*^	18	1.00 (0.00–1.75)	0
	**Number patients**	**Observed (%)**	
PVHI (present)	28	10 (36%)	None
Cystic changes (present)	28	8 (29%)	None

*PVHI, Periventricular Hemorrhagic Infarction*.

* *4th ventricle dilatation: 0-none, 1- compresses vermis, 2-compresses dorsal brainstem, 3-compresses ventral brainstem against clivus, 4- to supratentorial compartment*.

**Table 4:** NICU Network Neurobehavioral Scale (NNNS) measurements.

	**Cohort mean (SD)**	**Standardized relative to typical scores**	Typical mean^*^ (SD)
**MOTOR SUBSCORES**			
Excitability	3.43 (2.32)	−0.59 (−1.06 to 0.84)	4.23 (2.10)
Lethargy	4.79 (2.18)	−0.72 (−1.02 to −0.02)	6.32 (3.24)
Nonoptimal reflexes	4.96 (1.97)	0.39 (−0.18 to 0.97)	4.32 (1.73)
Asymmetric reflexes	1.41 (1.55)	−0.70 (−1.45 to 0.05)	1.93 (1.33)
Hypertonicity	0.29 (0.53)	−0.27 (−0.27 to 0.69)	0.07 (0.26)
Hypotonicity	0.64 (0.73)	−0.07 (−0.72 to 0.59)	0.55 (0.76)
Quality of movement	3.95 (0.86)	0.24 (−0.30 to 1.10)	3.81 (0.78)
**COGNITIVE SUBSCORES**			
Habituation	6.77 (1.82)	−0.80 (−2.26 to 0.08)	7.91 (1.14)
Attention	5.25 (1.16)	0.26 (−0.84 to 0.81)	5.30 (1.04)
Handling	0.47 (0.22)	0.85 (0.16 to 1.32)	0.27 (0.27)
Regulation	4.95 (0.74)	−0.28 (−0.74 to 0.67)	5.0 (0.82)
Arousal	3.63 (0.91)	−0.81 (−1.43 to 0.16)	4.16 (0.81)
Stress/ Abstinence	0.19 (0.05)	0.80 (0.20 to 1.50)	0.15 (0.05)

* *, per Maternal Lifestyle Study (Lester et al., 2011)*.

*SD, Standard deviation*.

**Figure 2:**
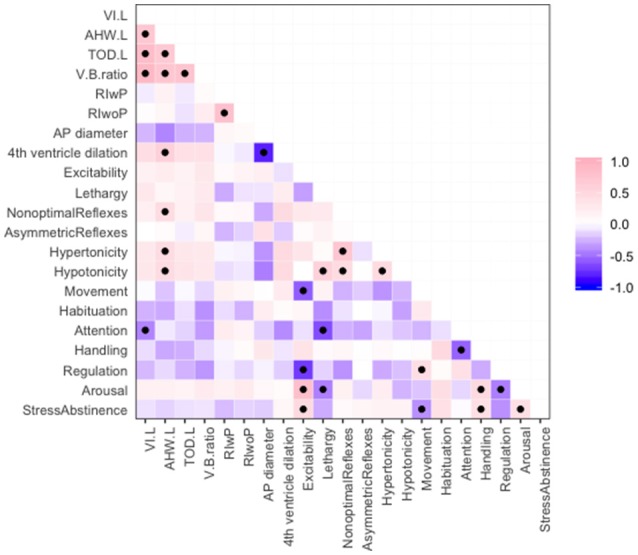
Correlations among ventricular size measurements and NNNS motor and cognitive subscores, where pink indicates positive correlation and purple negative correlation. Significant correlations with *p* < 0.05 are marked with a black dot.

Significant associations between imaging parameters and NNNS motor and cognitive subscores are summarized in Figure 2.

Abnormal radiology measures were associated with other abnormal radiology markers. Ventricle size measures (VI, AHW, TOD, and V/B ratio) were positively associated with one another (Figure 2). This is important as many studies have used different measurements to define ventriculomegaly (Sondhi et al., 2008; Maunu et al., 2011; Brouwer et al., 2012; Dorner et al., 2018). Larger AHW (but not other ventricle size measures) and larger fourth ventricle sizes were associated with smaller anterior-posterior diameter of the cerebellum (Figure 2). PVHI and cystic changes were associated; the odds ratio between PVHI and cystic changes was infinite (95% CI: 7.22—Infinity, *p* = < 0.001).

Features on both the HUS and NNNS exam were associated with receipt of neurosurgical intervention, where intervention was defined as either ventriculosubgaleal shunt (VSGS) placement or ventriculoperitoneal shunt (VPS) placement (Table 5). Fifty-Seven Percent of the cohort (16/28) required shunt placement; 2 patients required a VSGS, 11 had an initial VSGS followed by a VPS, and 3 patients had a VPS alone. Children receiving neurosurgical intervention were more likely to have larger ventricle sizes; means of ventricular size for those receiving neurosurgical intervention versus those not receiving intervention were statistically different as measured by: TOD (45.42 mm vs. 27.67, respectively, *p* = < 0.001), AHW (23.85 vs. 11.53 mm, respectively, *p* = 0.001), VI (24.62 vs. 14.20, respectively, *p* = 0.001), and V/B Ratio (0.615 vs. 0.378, respectively, *p* = 0.022) (Table 5). Neurosurgical intervention was most strongly associated with V/B ratio, with a correlation coefficient of 0.78 (Figure 2). The NNNS habituation subscore (response decrement to repeated auditory and visual stimuli) was the only additional parameter that differed between those infants receiving intervention and not receiving intervention (Table 5). Doppler RI values, with and without compression, did not correlate with receiving surgery.

**Table 5:** Means of HUS and MRI parameters, NNNS subscales by infants with and without neurosurgical intervention for hydrocephalus.

**Feature**	**Mean (SD) or *N* (%) for infants who received intervention** **(*n* = 16)**	**Mean (SD) or *N* (%) for infants with no intervention** **(*n* = 12)**	**Test for** difference (p^*^)
VI (mm)	23.88 (5.48)	12.58 (5.62)	<0.001^*^
AHW (mm)	22.88 (6.01)	9.75 (11.54)	0.003^*^
TOD (mm)	43.90 (9.00)	25.25 (14.95)	0.001^*^
V/B ratio	0.60 (0.08)	0.34 (0.08)	<0.001^*^
RI w/P	0.87 (0.11)	0.85 (0.10)	0.532
RI w/o P	0.83 (0.10)	0.79 (0.08)	0.209
AP diameter cerebellum (mm)	11.62 (7.43)	16.40 (3.65)	0.089
4th ventricle dilatation (mm)	1.31 (1.25)	0.60 (0.55)	0.116
PVHI (present)	8 (50%)	2 (17%)	0.114
Cystic changes (present)	7 (44%)	1 (8%)	0.088
Excitability	2.92 (1.98)	3.81 (2.54)	0.303
Lethargy	4.17 (1.70)	5.25 (2.44)	0.177
Nonoptimal reflexes	4.42 (1.51)	5.38 (2.22)	0.185
Asymmetric reflexes	1.17 (1.64)	1.60 (1.50)	0.487
Hypertonicity	0.17 (0.39)	0.38 (0.62)	0.286
Hypotonicity	0.50 (0.67)	0.75 (0.77)	0.371
Movement	4.06 (0.93)	3.87 (0.82)	0.569
Habituation	7.72 (1.68)	5.93 (1.57)	0.039^*^
Attention	5.50 (0.94)	5.08 (1.29)	0.359
Handling	0.54 (0.20)	0.43 (0.23)	0.217
Regulation	5.18 (0.76)	4.76 (0.71)	0.160
Arousal	3.46 (0.81)	3.75 (0.98)	0.403
Stress/abstinence	0.21 (0.05)	0.18 (0.06)	0.253

* *Significance determined by Student's t-test or by Fisher's exact test for categorical factors*.

Ventricle sizes were modestly correlated with motor abnormalities on NNNS exam (estimated correlations −0.72 to 0.39, Figure 2). Larger AHW sizes on HUS were associated with higher scores for nonoptimal reflexes (number of nonoptimal responses to reflex elicitation, such as excessive clonus), hypotonicity (number of hypotonic responses in arm, legs, trunk, or general tone) and hypertonicity (number of hypertonic responses in arm, legs, trunk, or general tone). Correlations between AHW and hypertonicity, hypotonicity, and nonoptimal reflexes subscores from the NNNS exam were: 0.59 (95% CI: 0.09–0.70, *p* = 0.018), 0.24 (95% CI: 0.08–0.70, *p* = 0.018), and 0.44 (95% CI: 0.08–0.70, *p* = 0.018), respectively (Figure 2).

Ventricle sizes were modestly inversely correlated with cognitive scores on NNNS exam as well (estimated correlations from −0.44 to 0.27, Figure 2). NNNS attention was negatively correlated with VI, where a larger VI was related to a lower average NNNS attention score (−0.06, 95% CI: −0.70 to −0.04, *p* = 0.034).

Of the 16 children in the cohort requiring intervention for posthemorrhagic hydrocephalus, 8 had antecedent Grade 3 IVH and 8 had PVHI (formerly called Grade 4 IVH). Presence of PVHI was associated with decreased or inadequate habituation to stimuli; the mean (standard deviation) for habituation for those with PVHI was 5.41 (1.75) and for no PVHI was 7.73 (1.18), *p* = 0.012.

There was clustering of NNNS exam findings in preterm children with IVH. In terms of motor development, those with abnormally high levels of motor, state, and physiologic reactivity (higher excitability scores) were more likely to have abnormal movement (quality of movement score), less regulation, and higher stress/abstinence and arousal scores. Those with low levels of activity, or lethargy, were more hypotonic, and exhibited lower attention and arousal scores. Interestingly, hypotonicity was correlated with hypertonicity (correlation 0.46, 95% CI: 0.11–0.71, *p* = 0.014) and both were correlated with nonoptimal reflexes: (hypertonicity—0.71, 95% CI: 0.46–0.86, *p* = 0.00) and (hypotonicity—0.45, 95% CI: 0.10–0.71, *p* = 0.015) (Figure 2).

NNNS scores were also associated with medical morbidities. Infants with severe ROP had higher NNNS Excitability [average (SD) 4.6 (1.9) vs. 2.3 (1.9), *p* = 0.006). Severe BPD was also associated with higher nonoptimal reflexes [5.4 (1.9) for those with BPD, 3.6 (1.6) without, *p* = 0.027] and lower movement [3.7 (0.8) for those with BPD and 4.7 (0.6) without, *p* = 0.003].

Of note, BPD does not confound the relationship of non-optimal reflexes with AHW; the mean AHW for infants without BPD is 14.4 mm and with moderate/severe BPD was 18.2, *p* = 0.308. No NNNS items were independently associated with Sepsis or NEC.

## Discussion

This is the first study to analyze the relationship between HUS and MRI imaging with neonatal neurobehavioral exams in posthemorrhagic ventricular dilation and hydrocephalus of prematurity. It is also the first to describe the range of NNNS findings in this group. Our patient population had severe ventriculomegaly, with means for all ventricular size measurements on HUS far above norms for age. As intended, the NNNS exams were performed around term equivalent age (average 37.3 weeks) in the attempt to correlate with published norms for term babies (Tronick et al., 2004). MRIs were also performed at term age equivalent (39.5 weeks), but only fourth ventricle dilation and anterior-posterior diameter of the cerebellum were analyzed due to technical challenges and lack of standardization of MRI reads for posthemorrhagic ventricular dilatation.

For this cohort of 28 preterm infants, the degree of ventriculomegaly—using precise imaging parameters such as V/B ratio and AHW—correlated with surgical intervention, as expected. This suggests that decisions regarding intervention are made either on the basis of ventricular size alone or due to changes in ventricular size in combination with the clinical course. Importantly, different radiologic measurements of the lateral ventricles were all related to each other, suggesting that the choice of ventricular measurement may be less important than choosing a standard measure. Specific ventricular measurements may be superior, however, for different purposes. In this study, intervention had the highest association with V/B ratio, AHW with NNNS motor subscores and the VI with NNNS cognitive subscores. The NNNS cognitive exam, specifically the habituation subscore, also correlated with receipt of surgical intervention. The relationship of intervention and NNNS exam suggests that posthemorrhagic hydrocephalus and/or intervention itself may create a detectable signal of reactivity.

In this participant group, V/B ratio best correlated with receipt of intervention. V/B ratios > 0.35 on ultrasound have been associated with smaller cerebrum and cerebellar volumes on term-corrected MRI (Govaert and de Vries, 1997). Decreased cerebral and cerebellar parenchymal volumes are associated with lower cognitive and language scores, abnormal motor outcome and processing speed (Nosarti et al., 2008; Maunu et al., 2009). Smaller cerebellar size, which has previously been shown to correlate with worse future cognitive outcomes (de Vries et al., 2019), was associated both with larger AHW and more fourth ventricle dilatation in our population.

Higher AHWs were associated with worse motor exams, specifically a higher number of nonoptimal reflexes and more hypotonicity. An increase in AHW has been suggested to be a more sensitive marker for early worsening of hydrocephalus and is seen subjectively as rounding of the frontal horns (Brouwer et al., 2010). Both lower (>6 mm) and higher (>10mm) AHW size cutoffs for surgical intervention have been used (Leijser et al., 2018; de Vries et al., 2019). The fact that early AHW dilatation correlates to exam findings in the neonatal period provides an opportunity to follow this parameter in a more multifaceted way.

Ventricle sizes and white matter injury were associated with worst neonatal cognitive assessment scores. Children with larger ventricles had lower attention scores; larger VI measurements were negatively correlated with the NNNS attention cognitive subscore. Attention is the ability to pay attention to salient stimuli in the environment. Additionally, PVHI, present in 10 of our children, correlated with decreased habituation to stimuli. Habituation is the ability to stop responding to (i.e., to ignore) repetitive stimuli. An inability to inhibit a response is one of the earliest measures of executive function and is well-developed by 6–7 months of age in typically developing infants (Diamond et al., 1994). In general, the children with larger VI and with PVHI were more irritable and responded more continuously to noxious stimuli, at the expense of paying attention to important stimuli such as a face or voice. It is possible that difficulty with attention and habituation in this early time frame are signs of later difficulty with inhibition and attention and a marker for later executive dysfunction (Diamond et al., 1994). Executive function and attention difficulties are well described in the hydrocephalus population (Holwerda et al., 2016). This early internal disorganization and over-responsiveness may be early markers of such difficulties.

Fourth ventricle dilatation on MRI was not associated with changes in NNNS exam (Table 5). While this attempt to evaluate fourth ventricle dilatation did not correlate with neonatal exams, this parameter has not yet been adequately assessed in this population. Further data are needed to understand the impact of fourth ventricular dilatation and on exams and outcomes.

We also saw informative patterns in the NNNS exam. Both ends of the arousal spectrum were seen; abnormally high excitability, stress and arousal score subgroups were present as were lethargy, hypotonia, and lower attention and arousal scores. These groups were not mutually exclusive; indeed, many children scored high on both lethargy and excitability, meaning during the exam they were often either too sleepy or too irritable. Both result in a failure to interact and pay attention to informative stimuli. Along the same lines, hypotonicity within our cohort was correlated with hypertonicity (correlation 0.46, 0.11–0.71, *p* = 0.014) and both were correlated with nonoptimal reflexes: (hypertonicity- 0.71, 0.46–0.86, *p* = 0.00) and (hypotonicity- 0.45 (0.10–0.71, *p* = 0.015). These patterns fit what we often see as a concerning pattern in ventricular dilatation; axial hypotonia and poor head control with high appendicular tone in the extremities. Together, these patterns of extreme reactions reflect a limited ability of the injured brain to regulate responses.

It is important to consider that ventricular dilatation and hydrocephalus are not isolated in preterm infants; the relationship of HUS and MRI measures to NNNS scores may be influenced by comorbidities. We found that severe ROP and moderate/severe BPD were associated with abnormalities in NNNS items regardless of ventricular size. Severe ROP was associated with higher NNNS excitability scores and moderate/severe BPD was associated with more nonoptimal reflexes and lower movement scores. Although both moderate/severe BPD and AHW were both associated with worse non-optimal reflexes, on further analysis BPD did not confound the relationship of non-optimal reflexes with AHW. It will be important in the future to consider both effects from hydrocephalus and comorbid conditions to better describe the contribution of hydrocephalus to neurodevelopmental delay.

Our data suggests that ventricular size is associated with neonatal exam as larger ventricle sizes were related to poorer scores on both motor and cognitive sections of the NNNS exam. The observed modest correlations are not unexpected; likely other factors in the infant's clinical course might also affect exam performance. Given that aspects of the neonatal neurobehavioral exam are associated with ventricular size measurements, our data suggests that exam and imaging may provide complementary and additive information on existing neurodevelopmental status.

There are limitations to the present study. Although a large portion of infants with posthemorrhagic hydrocephalus were captured, not all consecutive admissions to the NICU with IVH and some ventricular dilatation completed a NNNS evaluation; this may introduce selection bias. The average postmenstrual age at NNNS examination was 37.2 weeks, but some infants had exams earlier, as seen in Table 2. The nature of our NICUs are such that convalescing preterm infants, even after neurosurgery, are often transferred to lower acuity locations. As such, some infants were examined before term-corrected age due to imminent discharge. The impact of postnatal age on the NNNS is unclear in this study; previous studies have shown that postmenstrual age has an effect on NNNS subscores (Spittle et al., 2016). Additionally, given our small sample size, inclusion and analysis of all potential covariates was not feasible and we could not evaluate the cumulative impact of measured co-morbidities, such as NEC, on NNNS scores. Adequately powered larger samples are needed to address this important issue.

## Conclusion

In summary, for this cohort of 28 preterm infants with IVH and ventricular dilatation, surgical intervention was associated with degree of ventriculomegaly, using precise imaging parameters, as well as NNNS exam. Findings on both HUS and MRI imaging correlated with motor and cognitive abnormalities on neonatal neurobehavioral exam. These results suggest that larger neonatal ventricle sizes and white matter injury have detectable correlates on exam in this population. The NNNS exam may be additive with imaging in assessment of a common form of neonatal hydrocephalus. Importantly, the NNNS is identifying deficits at term equivalence, and even earlier, during a preterm infant's NICU stay. This risk identification is much earlier than typical neurodevelopmental exams done in follow-up care. This can allow for better developmental care during the NICU as changes can be made to the environment and rehabilitative interventions begun as soon as neurobehavioral deficits are identified (Mahoney and Cohen, 2005; Symington and Pinelli, 2006; King et al., 2008; Als and McAnulty, 2011; Fucile et al., 2011). It will be important to achieve longer-term follow-up in these infants to determine if these findings correlate with later neurodevelopmental disability.

## Ethics Statement

This study was carried out in accordance with the recommendations and approval of the Institutional Review Board at Johns Hopkins Hospital. Parents of all pediatric subjects gave written informed consent in accordance with the Declaration of Helsinki.

## Author Contributions

RD and VB conceived of and designed the study, performed exams, supervised data entry and analysis, analyzed and interpreted data, and wrote and edited manuscript. BS performed neuroimaging analysis. BS, JP, SR, and MA analyzed data and edited manuscript.

### Conflict of Interest Statement

The authors declare that the research was conducted in the absence of any commercial or financial relationships that could be construed as a potential conflict of interest.

## Abbreviations

AHW: anterior horn width
BPD: Bronchopulmonary dysplasia
HUS: Head ultrasound
IVH: Intraventricular hemorrhage
MRI: Magnetic Resonance Imaging
NEC: Necrotizing Enterocolitis
NICU: Neonatal Intensive Care Unit
NNNS: NICU Network Neurobehavioral Scale
PVHI: Periventricular hemorrhagic infarction
RI: Resistive Index
ROP: Retinopathy of Prematurity
TOD: Thalamooccipital Distance
V/B Ratio: Ventricular/Brain Ratio
VI: Ventricular Index
VPS: Ventriculoperitoneal shunt
VSGS: Ventriculosubgaleal shunt.
